# Low bone mineral density in men with chronic obstructive pulmonary disease

**DOI:** 10.1186/1465-9921-12-101

**Published:** 2011-08-03

**Authors:** James M Duckers, Bronwen AJ Evans, William D Fraser, Michael D Stone, Charlotte E Bolton, Dennis J Shale

**Affiliations:** 1 Section of Respiratory Medicine, Wales Heart Research Institute, School of Medicine, Cardiff University, University Hospital of Wales, Heath Park, Cardiff, CF14 4XN, UK; 2 Child Health, School of Medicine, Cardiff University, Heath Park, Cardiff CF14 4XN, UK; 3 Unit of Clinical Chemistry, School of Clinical Sciences, Liverpool University, Liverpool, UK; 4 Bone Research Unit, School of Medicine, Cardiff University, Academic Centre, University Hospital Llandough, Penlan Road, Penarth, Vale of Glamorgan, CF64 2XX UK; 5 NIHR Nottingham Respiratory Biomedical Research Unit, University of Nottingham, Clinical Sciences, City Hospital, Hucknall road, Nottingham. NG5 1PB. UK

**Keywords:** bone biomarkers, bone mineral density, chronic obstructive pulmonary disease, osteoporosis

## Abstract

**Background:**

Osteoporosis is common in patients with COPD but the likely multi-factorial causes contributing to this condition (*e.g*. sex, age, smoking, therapy) mask the potential contribution from elements related to COPD. In order to study osteoporosis and bone mineral density (BMD) related to COPD, we studied a well-defined group of patients and controls.

**Methods:**

BMD, forced expiratory volume in one second (FEV_1_), circulating bone biomarkers and biochemistry were determined in 30 clinically stable male ex-smokers with confirmed COPD and 15 age matched "ex-smoker" male controls. None of the patients were on inhaled corticosteroids or received more than one short course of steroids.

**Results:**

Mean (SD) FEV_1_% predicted of patients was 64(6)%, the majority having Global Initiative for Chronic Obstructive Lung Disease (GOLD) II airflow obstruction. There were 5/30 patients and 1/15 controls who were osteoporotic, while a further 17 patients and 5 controls were osteopenic. The BMD at the hip was lower in patients than controls, but not at the lumbar spine. Mean values of procollagen type 1 amino-terminal propeptide and osteocalcin, both markers of bone formation, and Type 1 collagen β C-telopeptide, a marker of bone resorption, were similar between patients and controls. However, all bone biomarkers were inversely related to hip BMD in patients (r = -0.51, r = -0.67, r = -0.57, p < 0.05) but did not relate to lumbar spine BMD. 25-OH Vitamin D was lower in patients.

**Conclusions:**

Men with COPD had a greater prevalence of osteoporosis and osteopenia than age matched male controls, with a marked difference in BMD at the hip. Bone biomarkers suggest increased bone turnover.

## Background

Chronic obstructive pulmonary disease (COPD) is a major cause of mortality worldwide [1]. In addition to progressive loss of lung function, there is an increasing awareness of the development of extra-pulmonary co-morbidities, and these include osteoporosis, cardiovascular disease and low skeletal muscle mass and function with an adverse effect on health outcomes [2].

A low bone mineral density (BMD), leading to osteoporosis is common in COPD with previous studies reporting osteoporosis in 24-44% of patients with COPD [3-7]. The aetiology of this loss is likely to be due to multiple factors including female sex, corticosteroid (CS) therapy, smoking, physical de-conditioning, vitamin D deficiency, hypogonadism and chronic systemic inflammation [3,4]. Although a low BMD is often asymptomatic, subsequent vertebral fractures may further compromise lung function [8,9], while hip fractures decrease mobility and increase the mortality risk [10].

Traditionally, loss of BMD, and osteoporosis in particular, have been considered "late manifestations" related to cumulative oral CS treatment of airways disease [11,12]. However, significant loss of BMD occurs in mild airways obstruction [4] and vertebral fractures have been reported in a high proportion of CS naive men with COPD [13]. That said, BMD is only one, albeit important, contributory cause of vertebral fractures, and other factors for *e.g*. heavy lifting, may play important roles. The impact of inhaled CS on bone status is unclear with conflicting findings in terms of the rate of loss of BMD, the risk of osteoporosis and the risk of fractures [14-20]. Many studies on the effect of inhaled CS are confounded by difficulties in quantifying the varying and often intermittent use of oral CS and few take in to account the potential for a disease specific component in BMD loss.

We hypothesised that osteoporosis would be present in men with COPD of mild to moderate severity airways obstruction and that this would be related to disease factors such as the persisting chronic systemic inflammatory state. We explored this hypothesis by determining BMD and circulating bone biomarkers in men with COPD and minimal/no CS exposure and no other secondary cause for osteoporosis.

## Method

### Study Subjects

Male ex-smokers with confirmed COPD were recruited at clinical stability; defined as no requirement for antibiotics or oral CS therapy and no change in respiratory symptoms beyond normal day to day variation in the preceding month [21,22].

Exclusion criteria for all subjects included a known diagnosis of or receiving treatment for osteoporosis, neoplastic disease or any disorder with an inflammatory or metabolic component, cardiac failure or requiring long-term oxygen therapy or on inhaled CS. Given that short course oral CS are occasionally given prior to diagnosis of COPD for "acute bronchitis", we pre-agreed and incorporated into the ethics proposal that one short course (< 1 week in duration) of oral CS would be allowed in a lifetime, which could be confirmed against primary care records. There were no other known secondary cause of osteoporosis.

Patients were recruited from 4 Cardiff and Vale GP surgeries covering a population of over 42,000 between them and 625 on their COPD registers. From the patients with COPD, only 53 (8.5%) met the study criteria and 23 of these were recruited. Patients were additionally approached at diagnosis of COPD during the study period from these surgeries (n = 5) and opportunistically from respiratory out-patients at University Hospital Llandough (n = 2) when the study criteria were met.

Healthy, sedentary, ex-smoker male control subjects free from respiratory symptoms and other exclusion criteria but with a minimum of 10 pack year exposure were also recruited from a database of past volunteers (n = 5) who had expressed a willingness to participate in future studies, as a spouse of out-patients (n = 5) and subjects attending smoking cessation clinics (n = 5).

All subjects gave written, informed consent and the study had Local Research Ethics Committee approval.

### Anthropometry, Lung Function and Incremental Shuttle Walk Test

Height and weight (Seca; Vogel and Halke, Hamburg, Germany) were determined barefoot and in lightweight indoor clothing and the body mass index (BMI) calculated. A low BMI was defined as < 20 kg/m^2 ^[4].

All subjects performed spirometry (FEV_1_, Forced Vital Capacity [FVC], and FEV1/FVC ratio), Vitalograph Ltd Bucks UK having withheld short acting and long acting bronchodilators for six and twelve hours respectively in accordance with ATS/ERS guidance [22]. Arterialised ear lobe gases were determined in patients seated at rest prior to exertion breathing air. Subjects also performed an incremental shuttle walk test to determine a distance (ISWD) following a practice attempt [23].

### Dual-Energy X-ray Absorptiometry (DXA)

Whole body composition and BMD at the lumbar spine and hip were determined by DXA (Hological Discovery, Hologic, Bedford, MA). The coefficient of variation (CV) was less than 2.2% for the lumbar spine, hip BMD, and fat-free mass (FFM). The FFM was expressed as a ratio to height squared to give an index: FFMI [4]. A low FFMI was defined as less than the lower 5^th ^percentile for the controls recruited for the study [4]. The BMD was expressed as an absolute value and as a T score (standard deviations from a young, sex-specific reference mean BMD) [24]. Osteoporosis was defined as a T score less than -2.5 for either the total lumbar spine, the total hip or each of the 3 hip subregions; osteopenia as T score less than -1 but greater than -2.5 [24].

### Bone turnover marker and biochemistry assays

An early morning, fasted venous blood sample was collected.

#### Bone turnover markers

Plasma biochemical markers of bone turnover were measured and all had inter and intra assay coefficients of variation of < 6.0% across the working range of the assays. Osteoprotegerin (OPG) was measured using a commercial enzyme linked immunosorbent assay (ELISA) (IDS Ltd Boldon UK) (detection limit 0.4 pmol/L).

##### Bone formation markers

Procollagen type 1 amino-terminal propeptide (P1NP): was measured using an ECLIA (Roche Diagnostics) (detection limit of 4 μg/L). Osteocalcin (OC) was measured using an electrochemiluminescence immunoassay ECLIA N-MID-OC (Roche Diagnostics Lewes UK)(detection limit of 0.6 ug/L).

##### Bone resorption marker

Plasma concentrations of β-C-telopeptides of type I collagen (βCTX) were measured using an ECLIA (Roche Diagnostics) (detection limits of 0.01 μg/L).

#### Biochemistry

Total testosterone, free thyroxine (fT4), thyroid stimulating hormone (TSH), insulin, total 25-OH Vitamin D (25 OH D) and parathyroid hormone (PTH) were all measured using direct competitive immunoassay (ADVIA Centaur and Diasorin Liason Analysers). Calcium (Ca), creatinine (Cr) and fasting glucose (FBG), total cholesterol (TC), high density lipoprotein (HDL), low density lipoprotein (LDL) and triglycerides were measured by standard methodology on the Abbott Aeroset (Abbott Diagnostics Berkshire).

#### Inflammatory mediators

Interleukin-6 (IL-6) was determined by immunoassay (Quantikine, R&D Systems Inc, MN, USA). Both intra- and interassay variation was < 10%, with a minimum detection limit of 0.70 pg/ml.

### Data Analysis

Data analysis was performed using the Statistical Package for the Social Sciences (SPSS, Chicago, IL), version 12.0. Log_10 _transformation was used where data was not normally distributed. Results are presented as arithmetic or geometric mean (for non-normally distributed) and standard deviation. Analyses included χ^2 ^test, independent t test, Pearson's correlations, one-way analysis of variance with post hoc Tukey analysis, and stepwise multiple regression analysis. A p < 0.05 was considered significant.

## Results

### Subject characteristics

The male patients (n = 30) and controls (n = 15) were matched 2:1 for age, Table 1. The patients had a greater pack year tobacco exposure, though the controls had between 10 and 80 pack years exposure. As expected, the patients had a lower FEV_1 _and shorter ISWD than the controls. Based on the Global Initiative for Chronic Obstructive Lung Disease (GOLD) severity criteria, [21] the patients comprised GOLD Stage I (n = 5), Stage II (n = 20) and Stage III (n = 5). No patients met the UK long-term oxygen therapy criteria (PaO_2 _< 7.3 kPa). Two patients had received a one week course of oral CS at 30 mg/day. No patients had been given inhaled CS; 6 were on long acting β_2 _agonist inhaler.

**Table 1:** Pulmonary Characteristics and Body Composition between Patients and Controls

	Controls (n = 15)	Patients (n = 30)	P value
**Age, yr**	63.5 (5.7)	66.0 (8.5)	0.25

**Smoking pack-years, median (range)**	34.3 (10-80)	53.3 (10-150)	0.04

**Long acting β_2 _agonist n**	0	6	

**FEV_1 _(% predicted)**	92.9 (10.6)	63.7 (17.9)	< 0.001

**FEV_1_(l)**	2.88 (0.48)	1.95 (0.63)	< 0.001

**FVC (l)**	3.80 (0.71)	3.41 (0.84)	0.12

**O_2 _saturations at room air (%)**	96.3 (1.7)	95.4 (1.7)	0.08

**pO_2 _(kPA)**	ND	9.64 (1.5)	

**ISWD (m)**	563 (221)	424 (171)	0.03

**BMI (kg/m2)**	29.7 (3.5)	26.4 (5.1)	0.03

**Total FFMI (kg/m^2^)**	20.4 (1.7)	18.3 (2.4)	0.004

**BMD Total Hip (g/cm^2^)**	1.05 (0.17)	0.93 (0.14)	0.01

**BMD Femoral neck (g/cm^2^)**	0.85 (0.14)	0.74 (0.11)	0.01

**BMD Total lumbar spine (g/cm^2^)**	1.13 (0.23)	1.03 (0.20)	0.11

Data presented as means (SD) unless stated otherwise

Definition of abbreviations: FEV_1 _= Forced expiratory volume 1 second, FVC = Forced vital capacity, RA = Room Air, ISWD = Incremental Shuttle Walk distance, BMI = Body Mass Index, BMD = Bone Mineral Density, FFMI = fat-free mass index, FMI = fat mass index, O_2 _= oxygen, ND = Not determined

### Body Composition

The BMI was less in the patients than controls and three of the 30 patients had a low BMI compared with none of the controls, Table 1. The patients with a low BMI also had low FFMI and six other patients had a low FFMI with normal BMI-hidden loss of FFM. Only one control subject had a low FFMI.

### Bone Mineral Density

Total BMD at the hip and also at the three hip sub regions was lower in patients than controls (p < 0.05) while the BMD at the lumbar spine was not different, Table 1. In patients both FFMI (r = 0.51, p = 0.004) and FEV_1 _(r = 0.51, p = 0.004) were related to the hip BMD but not to lumbar spine BMD.

Within the patient group, multiple regression analyses were performed with either total BMD at the hip or lumbar spine as the dependent variable, and age, smoking pack years, FEV_1_, and FFMI as independent variables. The FEV_1 _and FFMI (p < 0.05) were both predictive for hip BMD with an adjusted R^2 ^= 0.35, Table 2. At the lumbar site, smoking pack year history (p < 0.05) was predictive for BMD with an adjusted R^2 ^= 0.12.

**Table 2:** Multiple Regressions for Bone Mineral Density in Patients

	R^2^	β	SE	P
**BMD total Hip**				

**Constant**		0.331	0.166	0.056

**FEV_1_**	0.23	0.087	0.035	0.019

**FFMI**	0.12	0.023	0.009	0.02

**BMD total Lumbar spine**				

**Constant**		1.159	0.068	< 0.001

**Smoking pack years**	0.12	-0.002	0.001	0.035

Definitions of abbreviations:- BMD = Bone Mineral Density; FEV_1 _= Forced expiratory volume 1 second; FFMI fat-free mass index;

### Osteoporosis and Osteopenia

**Figure 1:**
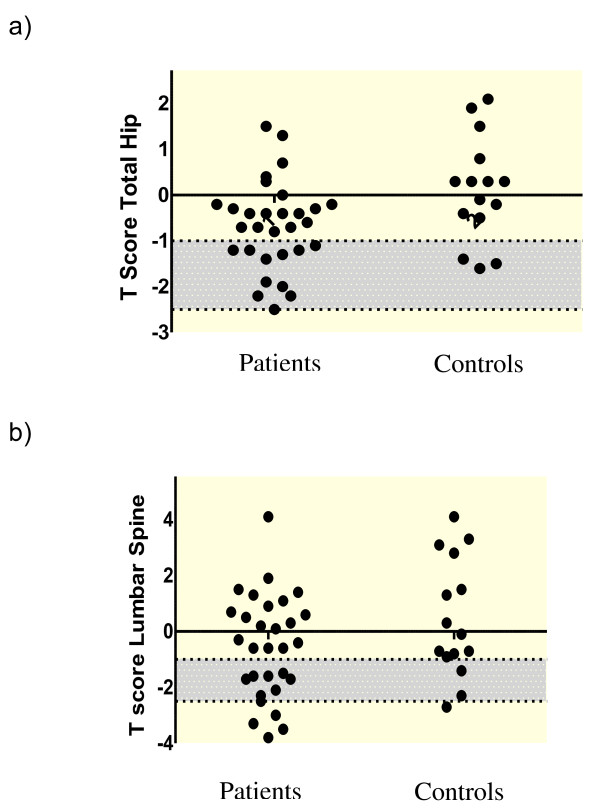
**The T Score at the Hip and Lumbar Spine**. Figure 1a T score Total Hip. Figure 1b T score Total Lumbar Spine. Shaded grey area represents osteopenia (T score -1.0 to -2.5). Osteoporosis below T score -2.5

Five patients (17%) had osteoporosis at either site - two had osteoporosis at the total hip or a sub region site and five had osteoporosis at the lumbar spine, Figure 1a and 1b. Importantly, the 2 patients who had previously received a one week course of oral CS in their lifetime were not osteoporotic.

One (7%) control subject had osteoporosis at the lumbar spine. Seventeen (57%) patients and five (33%) controls had osteopenia - predominantly in the hip in both subject groups.

### Bone Biochemistry

Circulating biochemical markers of bone formation (P1NP and OC) and resorption (βCTX) were similar between patients and controls, Table 3. There was no association between any biochemical bone turnover marker and smoking pack year history or FFMI. In the whole group there was an association between log_10_P1NP and log_10_OC (r = 0.81, p < 0.001) and log_10 _βCTX (r = 0.85, p < 0.001).

**Table 3:** Biochemistry and Markers of Bone Metabolism

	Controls (n = 15)	Patients (n = 30)	P value
**25 OH Vitamin D (ug/l) #**	16.1 (1.4)	11.4 (1.9)	**0.03**

**PTH (pmol/l)#**	6.71 (1.36)	6.12 (1.74)	0.58

**Adjusted Calcium (mmol/l)**	2.32 (0.09)	2.37 (0.11)	0.14

**Creatinine (μmol/l)**	100 (14)	95 (19)	0.36

**Fasting glucose (mmol/l)**	5.7 (0.7)	5.4 (0.6)	0.07

**Testosterone (nmol/l)**	13.9 (4.1)	14.8 (6.2)	0.62

**T4 (pmol/l)**	14.99 (1.97)	15.37 (2.47)	0.60

**TSH (mU/l)**	1.72 (0.91)	1.76 (0.81)	0.89

**IL-6 (pg/ml) #**	3.1 (2.2)	4.8 (2.8)	0.28

**OPG (pmol/l) #**	6.5 (1.9)	8.5 (1.4)	0.06

**Osteocalcin (μg/l) #**	23.0 (1.4)	22.7 (1.5)	0.93

**P1NP (μg/l) #**	40.9 (1.4)	45.6 (1.9)	0.47

**CTX (μg/l) #**	0.4 (1.5)	0.4 (1.9)	0.69

Data presented as mean (SD), unless # Geometric Mean (SD)

Definition of abbreviations:- PTH = parathyroid hormone, T4 = free thyroxine, TSH = thyroid stimulating hormone, IL-6 = Interleukin -6, OPG = osteoprotogerin, P1NP = Procollagen type 1 amino-terminal propeptide, βCTX = β-C-telopeptides of type I collagen

**Figure 2:**
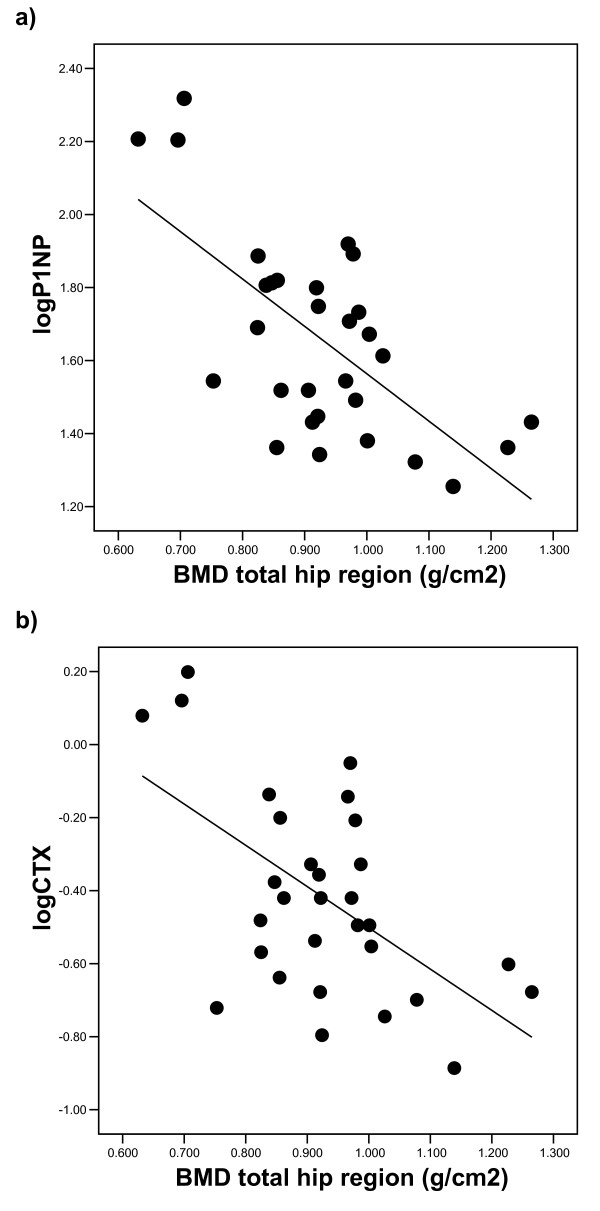
**The relationship of the BMD at the hip to bone biomarkers in Patients**. Figure 2a The Marker of Bone Formation P1NP. Figure 2b The Marker of Bone Resorption CTX.

The hip BMD in patients was inversely related to log_10_P1NP (r = -0.67), log_10_OC (r = -0.51), and log_10 _βCTX (r = -0.57), all p < 0.05, Figure 2a &2b. Further, hip BMD was related to log_10_OPG (r = -0.41). All of these associations persisted if adjusted for age and smoking pack years. Similar associations remained if the hip T score was substituted for absolute hip BMD, but there were no relationships with either measure at the lumbar spine. Circulating biomarkers of bone formation and resorption were not related to BMD at the hip or lumbar spine in the controls.

OPG was greater in osteoporotic than non-osteoporotic patients (p < 0.05) when corrected for age. Other bone markers were not different between osteoporotic and non-osteoporotic patients.

Mean 25-OH Vitamin D were lower in patients, Table 3. Insufficient total 25-OH Vitamin D levels (< 20 μg/L) were recorded in 24 (80%) of the patients and nine (60%) of the controls, χ^2 ^= 0.26 [25]. Of these, nine patients and one control had significantly low total 25-OH vitamin D levels (< 8 μg/L).

PTH above the reference range, all with normal adjusted calcium and creatinine levels, were recorded in 13 (43%) patients and three (20%) controls. Elevated PTH levels in conjunction with significantly low total 25-OH Vitamin D, but normal adjusted calcium levels were seen in four (13%) patients and one (7%) control.

Neither log_10 _total 25-OH Vitamin D or log_10_PTH were associated with BMD at the hip or lumbar spine or any of the biochemical markers of bone turnover in the whole group or patient subset (p > 0.05).

### Other Biochemistry and Systemic inflammation

Thyroid function tests were normal in all subjects and there was no difference in fT4 and TSH between patients and controls. Total testosterone levels was not different between patients and controls, however four (13%) patients and two (13%) controls had low early morning levels (< 8.0 nmol/L). Circulating IL-6 was similar in patients: 4.8 (2.8) and controls: 3.1 (2.2) pg/ml, p = 0.28. There was no difference in IL-6 between those patients with and without osteoporosis, p = 0.45. Nor was log_10_IL-6 related to BMD at the hip or lumbar spine.

## Discussion

Men with COPD had a low BMD, with a greater prevalence of osteoporosis at the hip and lumbar spine, compared with age matched, ex-smoker, sedentary, male controls. This highly select group of males were pre-defined to remove the possible confounding effects of the female post-menopausal loss of BMD and additionally with no/minimal oral CS to eliminate the contribution of these agents to bone thinning. Further, most had mild severity airways obstruction with over 80% of the men with COPD being in GOLD class I or II. Thus, our findings in this group of patients, suggest that the traditional view of bone thinning and osteoporosis occurring largely as a result of CS use is simplistic. Our findings could be interpreted as indicating a disease specific component involved in the loss of BMD in COPD.

We confirmed the previous relationships between both BMI and FFMI with BMD in COPD [4,26]. The FFM is a surrogate of peripheral skeletal muscle mass and was reduced in nine of our patients, including six with a normal BMI. This suggests preferential skeletal muscle mass loss in 20% of our patients, which is in keeping with levels reported in patients with more severe lung disease. The importance of the link between FFMI and BMD status was emphasised by the predictive nature of this variable along with airways obstruction for hip BMD [4]. This suggests that even in milder severity airways obstruction there are the same relationships between muscle mass loss and bone thinning as previously reported in more severe status patients [4,26]. It is unclear from this study what the causative link is, but we previously demonstrated a parallel increased excretion of cellular protein and bone collagen breakdown products in patients with a low FFM and BMD indicating a protein catabolic state in COPD, which may be a factor linking bone and skeletal muscle mass loss [4]. Further, the association of severity of emphysema on CT scan to low BMD was emphasised in 2 recent studies, one of men with COPD and the other comprising of tobacco exposed individuals, 60% having COPD [27,28], again suggesting a systemic proteolytic effect.

Within our patient group there are other possible causes of bone thinning including physical inactivity. The shorter ISWD in the patients indicates a reduced functional capacity for exercise [23] despite their airways obstruction being relatively mild. This reduction even in milder airways obstruction, an interpretation supported by a similar observation [29], may contribute to physical deconditioning, which might exert a greater effect on hip BMD than on the lumbar spine and possibly explain the differences we report between the two sites. Differences between the hip and lumbar spine sites may also be due to loss of skeletal muscle mass from the lower limbs and reduction in weight bearing activity as well as differences in bone composition between the hip and lumbar spine.

Other potential factors in the loss of BMD are changes in bone homeostasis due to alterations in hormone and vitamin activity and systemic inflammation. In health, bone is metabolically active with continuous remodelling as an adaptation to changes in distribution of mechanical force and to repair damage. Bone resorption and formation are normally tightly coupled, but loss may occur if this balance is disturbed. Currently bone homeostasis in COPD is not fully understood. We therefore explored the use of circulating biomarkers of bone formation and resorption in this study as they have been widely used in non-COPD osteoporosis as indicators of bone turnover [30]. They provide insight into bone physiology and pathophysiology, and have been used to monitor the response to the treatment of osteoporosis in post-menopausal women [31]. However, to date there is little experience of their use in COPD, having only been studied in a small pre-transplant population [32]. We found no difference between the mean levels of any of the bone biomarkers in patients and controls. However, in patients, the greatest levels of both bone formation and resorption markers were associated with a low BMD at the hip. This may indicate that increased bone turnover accounts for altered BMD at the hip. This is similar to the pattern of bone biomarkers seen in the majority of post-menopausal women with osteoporosis [33].

Chronic systemic inflammation has been postulated as a mechanism in the loss of BMD in COPD, but there was no difference between patient and controls for IL-6, which has been implicated with TNF-a in post-menopausal osteoporosis and which *in vitro *stimulates osteoclasts and bone resorption [34]. However, systemic levels are unlikely to reflect this tissue level and we have previously been unable to relate BMD to systemic levels in other patients with COPD. Interestingly, OPG was greater in osteoporotic patients than those without osteoporosis and related inversely to hip BMD. It could be considered as a pro-formation marker due to its potential to act as a decoy for receptor activator for NF kappa B ligand (RANKL) and thus act as a local anti-inflammatory agent. However, caution is needed in interpreting the OPG level and the ratio of OPG to RANK-L may be more informative but at present there is debate about the sensitivity of the RANK-L assays available.

In pre-transplant patients with COPD Forli et al [35] reported a direct relationship between TNF-α receptor II and the resorption marker βCTX, whilst Bon et al analysed 27 inflammatory mediators in relation to several serological markers of bone turnover in patients with severe COPD pre transplant, encompassing patient on OCS and osteoporosis treatment [32]. The lack of a relationship between IL-6 and bone turnover biomarkers in our study mirrors previous studies [4] but may be a result of the relatively mild airways obstruction of our patients and the small population size.

The PTH, Cr, adjusted Ca and total testosterone levels, and thyroid function, all of which are involved in bone homeostasis were not different between patients and controls, though, the patients had a lower total 25-OH Vitamin D level than controls confirming previous findings [36]. Insufficient total 25-OH Vitamin D levels were seen in 80% of patients with around a third having significantly low levels. Low levels may reflect both a disease related component, such as decreased physical activity [29] and lower sunlight exposure, and the generally low levels reported in aging populations. Indeed, 60% of the control subjects had insufficient 25-OH Vitamin D levels. In four patients with insufficient 25-OH Vitamin D and increased PTH there may have been some osteomalacia, but we did not differentiate osteomalacia from osteoporosis [37].

In this study, we did not quantitatively assess for vertebral fractures in COPD, though marked distortion on the DXA images excluded that specific vertebra from evaluation - in keeping with standard clinical evaluation of lumbar vertebrae, n = 5 individual vertebrae. A recent paper retrospectively reviewed men diagnosed with COPD, picking up a high rate of fragility fracture but a low proportion having a DXA or being treated with anti-resorptive therapy[38]. The EOLO study reported an increased risk of fracture with increasing severity of airflow obstruction in addition to the contribution of daily inhaled steroid use [20]; with McEvoy demonstrating a high fracture rate in steroid naïve men [13].

The limitation of this study is the modest size of the study population. Recruiting men with COPD who met the rigorous study entry criteria proved challenging despite working with across several primary care surgeries and screening large numbers of patients. The proportion of male controls with osteoporosis mirrored our previous research in two different male control populations of a similar age that we have studied, though osteoporosis was not reported in these publications according to gender [4,5]. The controls and patients were not matched for smoking history despite attempts, though the controls had a median 34 pack year tobacco exposure.

The measurement of BMD using DXA may be confounded by several factors [39]. In particular, measurement of the lumbar spine BMD using DXA becomes less useful with increasing age due to confounding effects such as vertebral collapse, osteophytes and aortic calcification all leading to a "pseudo-normalising" of the BMD [40].

## Conclusion

A low BMD occurs in males with COPD of mild to moderate severity airways obstruction, with minimal/no CS exposure compared to ex smoker controls and osteoporosis is common. This study suggests a disease related causative component. As in previous studies, a low BMD was more prominent at the hip compared with the lumbar spine, which may reflect physical deconditioning or different bone composition. The mechanism of BMD loss in COPD remains unclear but our results suggest increased bone turnover. Our findings highlight the potential value of studying milder severity patients free from potential causative confounders and reinforce the need for earlier identification and targeting of risk factors for osteoporosis as part of the management of COPD.

## Abbreviations

BMD: Bone mineral density; P1NP: Procollagen type 1 amino-terminal propeptide; CTX: β-C-telopeptides of type I collagen

## Competing interests

The authors declare that they have no competing interests.

## Authors' contributions

JD helped design the study, conducted the clinical assessments, analysed and interpreted data and wrote the first draft; BE helped design the study and contributed to the interpretation and writing; WF contributed to the interpretation and writing; MS contributed to the interpretation and writing; CB helped design the study and contributed to the interpretation and writing; DS helped design the study and contributed to the interpretation and writing. All authors have read and approved the final manuscript.

## Funding

Dr J Duckers was supported by a Cardiff and Vale NHS Trust Clinical Research Fellowship. Dr C Bolton is currently funded by the NIHR Nottingham Respiratory Biomedical Research Unit.
